# Static and dynamic functional connectivity supports the configuration of brain networks associated with creative cognition

**DOI:** 10.1038/s41598-020-80293-2

**Published:** 2021-01-08

**Authors:** Abhishek Uday Patil, Sejal Ghate, Deepa Madathil, Ovid J. L. Tzeng, Hsu-Wen Huang, Chih-Mao Huang

**Affiliations:** 1 Department of Sensor and Biomedical Technology, School of Electronics Engineering, Vellore Institute of Technology, Vellore, Tamil Nadu India; 2 Department of Biological Science and Technology, National Chiao Tung University, Hsinchu, Taiwan; 3 Center for Intelligent Drug Systems and Smart Bio-Devices (IDS2B), National Chiao Tung University, Hsinchu, Taiwan; 4 Cognitive Neuroscience Laboratory, Institute of Linguistics, Academia Sinica, Taipei, Taiwan; 5 College of Humanities and Social Sciences, Taipei Medical University, Taipei, Taiwan; 6 Department of Educational Psychology and Counseling, National Taiwan Normal University, Taipei, Taiwan; 7 Hong Kong Institute for Advanced Study, City University of Hong Kong, Kowloon, Hong Kong; 8 Department of Linguistics and Translation, City University of Hong Kong, Kowloon, Hong Kong

**Keywords:** Computational neuroscience, Cognitive neuroscience

## Abstract

Creative cognition is recognized to involve the integration of multiple spontaneous cognitive processes and is manifested as complex networks within and between the distributed brain regions. We propose that the processing of creative cognition involves the static and dynamic re-configuration of brain networks associated with complex cognitive processes. We applied the sliding-window approach followed by a community detection algorithm and novel measures of network flexibility on the blood-oxygen level dependent (BOLD) signal of 8 major functional brain networks to reveal static and dynamic alterations in the network reconfiguration during creative cognition using functional magnetic resonance imaging (fMRI). Our results demonstrate the temporal connectivity of the dynamic large-scale creative networks between default mode network (DMN), salience network, and cerebellar network during creative cognition, and advance our understanding of the network neuroscience of creative cognition.

## Introduction

The burgeoning field of network science and its applications to cognitive and clinical neuroscience has provided a framework to help understand the complex neurocognitive functions and reveal the pathological basis of neurological disorders. A network science approach helps us to understand the brain as a network through local and distributed processes^1–4^. Such an approach has been used in functional human neuroimaging (e.g., functional magnetic resonance imaging, fMRI) studies to understand Alzheimer’s diseases^5–9^, epilepsy^10,11^ and schizophrenia^12–14^, and to investigate cognitive functions associated with learning^15^, behavior^16^ and task performance^17,18^. The relational and causal association of distributed brain regions with various cognitive functions have been mapped to reveal the connectome of the human brain^19,20^.

The neuroscientific literature mostly considers domain-general functions for which multiple regions and functions are engaged, resulting in diverse quantifications^19,21^. However, little has been reported of domain-specific cognitive functions associated with specific brain networks^22,23^. It is important to understand the domain-specific functions in the brain that result in activations in the specific brain regions^15,23^. Brain networks and the functional connections between brain regions involved in task execution behave dynamically with respect to time. Therefore, there is a need to explore the functional nature of cognition by investigating dynamic and domain-specific brain networks. This brain state during cognition can be uncovered by assessing connectivity in either static or dynamic brain networks. Most neuroimaging studies represent static brain networks over the entire duration of an fMRI experiment^24–26^. These networks represent the functional connectivity and interactions between various cortical and subcortical regions of the brain over this task-related period. However, activity in static networks does not clearly show the changes that occur over short durations during the fMRI scan; hence, there is a need to develop dynamic reconfiguration-based methods to identify the modular architectures of evolving networks^15,27,28^. Minute-by-minute changes in neuronal activities or blood flow changes in the order of milliseconds result in dynamic re-configuration^29,30^. Dynamic reconfigurations can be analyzed by dividing a task-based fMRI scan session^15,27,28^ into time windows which are further used to understand the interactions between brain regions. Such dynamic reconfigurations are more sensitive than the static re-configurations in their ability to detect changes in cognition states^17^.

There have been significant developments of various neuroscience methods and protocols for understanding creative-cognition^31–33^. Previous studies have revealed connectivity patterns within various creativity-related brain regions, in particular in the default mode network (DMN) and the executive control network^34,35^. The DMN has been suggested to cooperate with other networks during creative cognition, such as episodic and semantic memory retrieval, mind wandering and idea generation^36–41^. Convergent thinking is the mental ability to find a common solution to a particular problem at hand by recruiting various cognitive processes^42,43^. These processes have been suggested to involve strategic thinking that narrows down the ideas to reach the correct solution^35,44,45^. The most popular task representing convergent thinking is the remote associate’s test (RAT)^46–48^, which indicates the associative ability^42,49,50^. Greater associative ability has been seen in more creative people^51^ and individuals with greater associative ability have been shown to have more complex connectivity within their brain networks associated with creative cognition^52,53^. EEG and fMRI studies have explored varied perspectives related to the RAT^54,55^. Studies have found consistent patterns of associations in the superior temporal gyrus during convergent thinking tasks^56,57^. These activations were co-related to intuition associated with activity in the right anterior region of the superior temporal gyrus. A recent meta-analysis suggests that the left inferior frontal gyrus, right medial frontal gyrus, hippocampus and the amygdala are also involved in solving problems creatively^58^. However there has been no investigation of the relationship between creative thinking tasks such as the RAT and dynamic reconfigurations in the light of brain networks associated with creative cognition.

The field of dynamic network neuroscience has recently begun to examine the dynamics of integrative nature of brain networks interactions involved in specific cognitive processes^15,59,60^. This emerging approach of temporal dynamics uses networking^60,61^ to underpin the evolution of these dynamic networks and link these connections to behavioral outcomes. Community detection based on graph theory is one of the most important and widely used metrics to understand the interaction, organization, and evolution of functionally connected brain networks^62^. A community is a set of densely interconnected brain regions(i.e., showing network integration) and loosely connected to other communities (i.e., showing network segregation)^63,64^. Community detection has been used in several neuroimaging studies of structural and functional brain networks^65–67^. Most of the fMRI studies have explored intrinsic functional connections assuming a static re-configuration in the networks^68–72^. Recent neuroimaging studies have used this new and promising approach and investigated dynamic network reconfigurations to assess the temporal changes in the communities in large and complex brain networks^15,69,73^.

**Figure 1:**
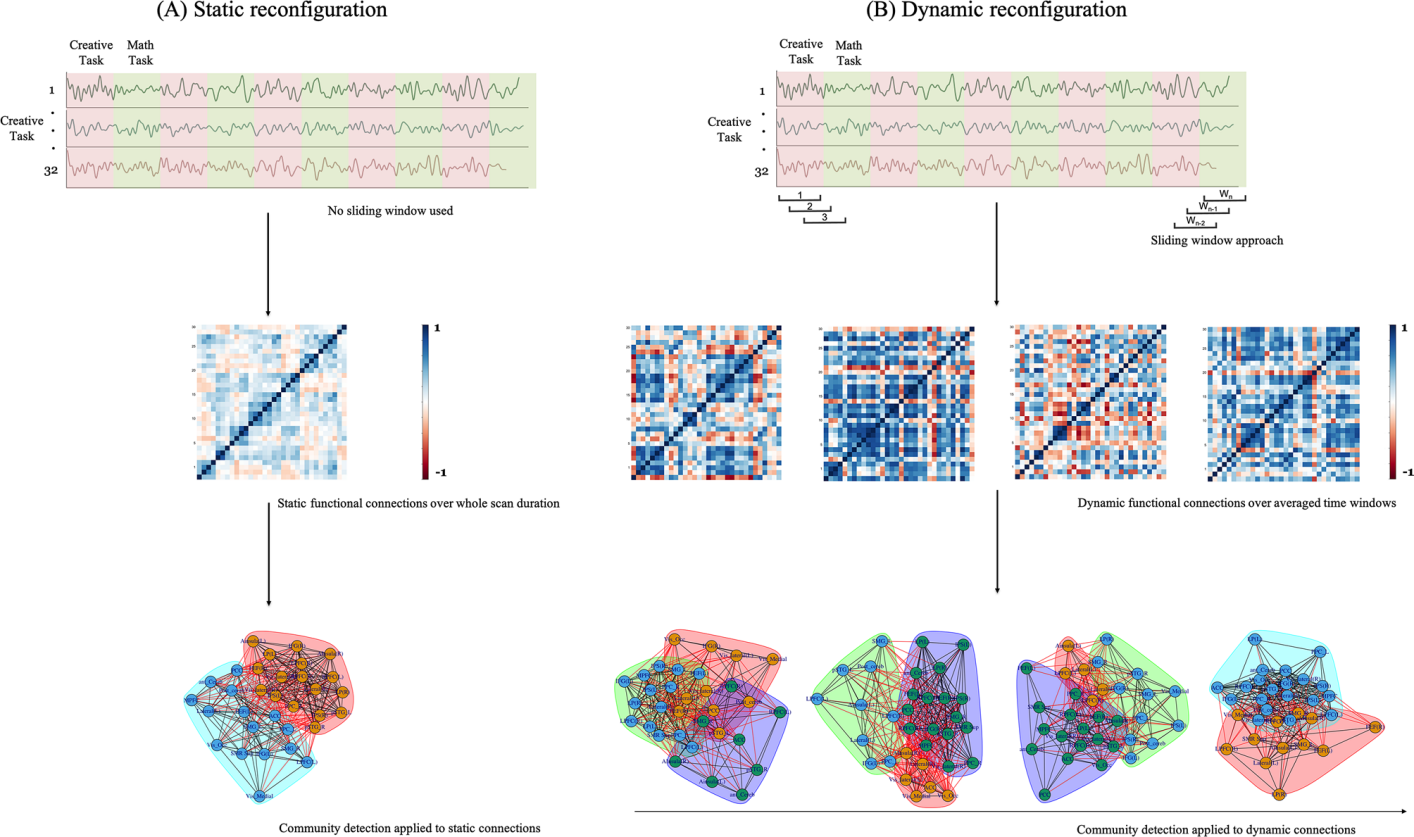
Overall flow of the static and dynamic network reconfiguration during creative thinking task. (**A**) Static reconfiguration. The Blood oxygen level dependent (BOLD) time series signal was extracted from each of the 32 regions of interests (ROIs). The z-scored Pearson correlation coefficients for 32 ROIs were calculated and a statistical threshold of *p* < 0.05, false discovery rate (FDR)-correction was applied to obtain the estimated correlation map. A single adjacency matrix was estimated and a community detection algorithm was applied to identify communities for the whole brain flexibility over the entire scan duration. (**B**) Dynamic reconfiguration. The Blood oxygen level dependent (BOLD) time series signal was extracted from each of the 32 regions of interests (ROIs). An overlapping sliding window (length W) was used and time ordered functional connectivity matrices were estimated over these windows for all the ROIs using z-scored Pearson correlation. The estimated correlation maps W_n_ (n is number of windows) were statistically thresholded with *p* < 0.05 (FDR correction). A community detection algorithm was applied to all these matrices to detect communities across the windows.

In the present study, we examined the static and dynamic configuration and evolution of brain networks associated with creative cognition in young adults performing an event-related creativity fMRI experiment. We also aim to compare these results of the modified Chinese version of the remote associates task (CAT) which was used to demonstrate the dynamic interaction between different brain regions to support complex cognitive processes during a creativity task with the resting-state condition. The CAT was adopted from Mednick’s (1962) Remote Associates Task (RAT) and its reliability and validity have been confirmed to be as effective as the RAT in measuring the processes of creative thinking^74^. The brain network interaction in this creative thinking specifically is integrated with goal-directed memory retrieval and prepotent-response inhibition of semantic information^35^, providing rich spatial and temporal dynamics of brain function. We were particularly interested in examining the functional interactions and temporal variability between various brain regions associated with creative cognition and at rest. First, the network was parcellated into 32 seed regions of interest (ROIs) that correlated with 8 commonly known networks^75^. We then examined the static and dynamic interactions which were not only network-specific but also region-specific for creative task sessions and the resting-state session. We applied the sliding window approach for the dynamic interaction analysis and extracted the dynamic temporal networks from the blood-oxygen level dependent (BOLD) signal of the ROIs to form the basis of these complex brain networks. The sliding window divided the BOLD signal of the 32 seed ROIs into time-varying windows. These windows represent task-related functional and temporal connections and interactions within these brain networks associated with creative cognition. To understand these connections and interactions, Pearson correlations were obtained for the entire fMRI scan period representing the static brain network and for the temporal windows representing the dynamic brain networks. This was obtained for both creative tasks as well as resting-state sessions. The general flow of methods for the static and dynamic reconfiguration in this study is depicted in Fig. 1.

Detecting various communities and dividing the network into sub-networks by maximizing the modularity function is an important feature of network analysis^63,76,77^. To understand the variation in static network, dynamic networks, creative task and rest we used a popular and fast iterative algorithm called the fast greedy community detection algorithm from previous fMRI studies^78–81^. This algorithm uses a hierarchical and bottom-up approach to optimize the modularity function in detecting communities within complex brain networks^82–84^. The assignment of a community to a particular node in a network is achieved by a statistically quantified modularity function^85–88^. We applied the community detection algorithm to the dynamic temporal windows to understand the evolution of the complex brain networks associated with creative cognition. We also characterized an important dynamic brain network measure that we call “flexibility” to understand the adaptations of dynamic brain networks associated with creative cognition^15^.

## Results

### Head motion considerations

To estimate the potential influence of head motion on the functional connectivity analysis, the mean head motion (mm) and maximum head motion (mm) were assessed using frame wise displacement (FD) across resting-state and creative task fMRI data^89^. The values of mean FD across three fMRI sessions are as follows: resting-state fMRI session = 0.016 (SD = 0.006), creative task fMRI 1st session = 0.149 (SD = 0.055), creative task fMRI 2nd session = 0.180 (SD = 0.070). The values of Power’s mean FD^90^ across three fMRI sessions are as follows: resting-state fMRI session = 0.018 (SD = 0.007), creative task fMRI 1st session = 0.176 (SD = 0.068), creative task fMRI 2nd session = 0.217 (SD = 0.094). The correlation analyses performed between mean FD, Power’s mean FD and BOLD time series for each ROI for resting-state fMRI session, creative task fMRI 1st session, and creative task fMRI 2nd session also showed the minimum of influence of head motion (refer Supplementary Table S1). These results suggest that the results of functional connectivity in this study were unlikely to be driven by motion confounds.

### Task and rest static functional connectivity results

**Figure 2:**
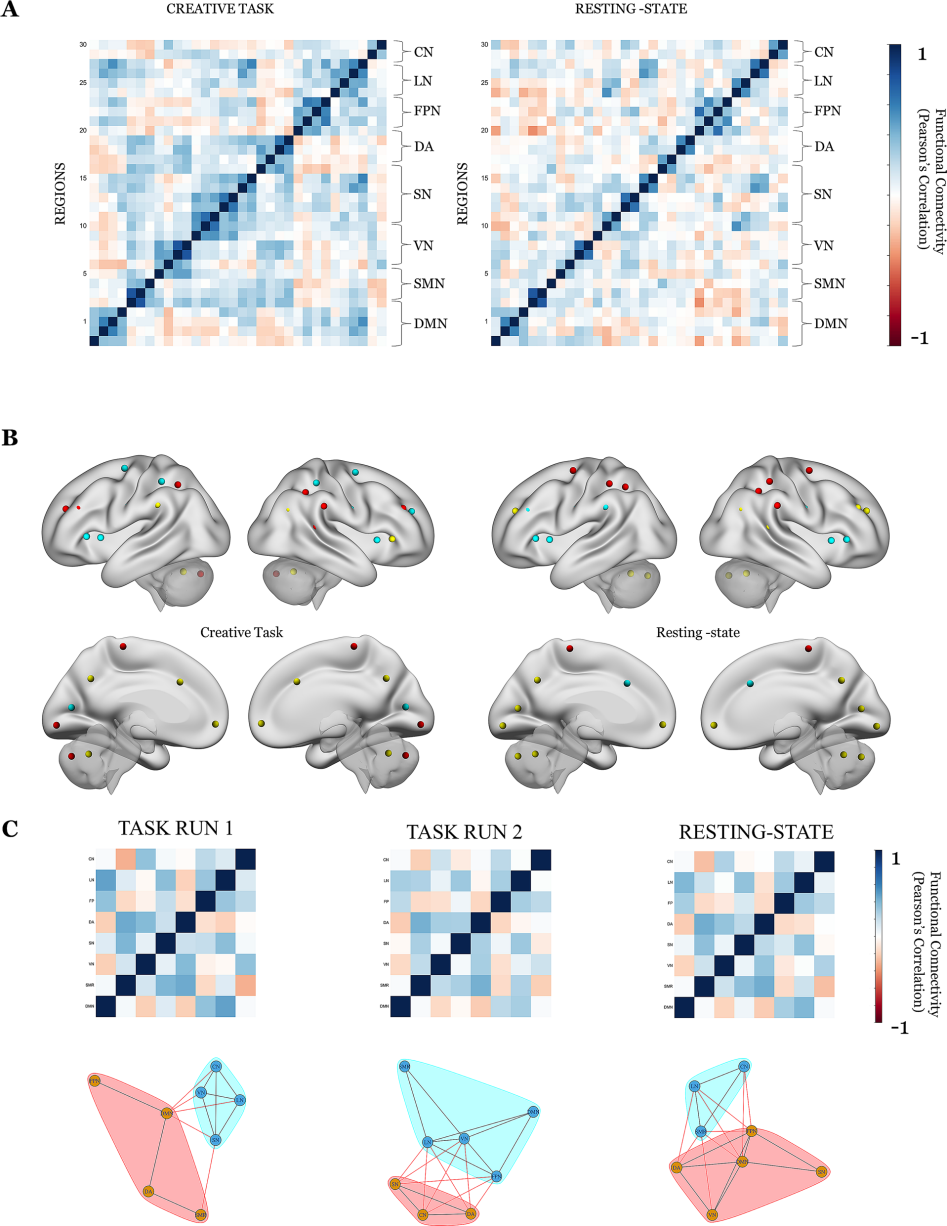
Comparison of the creative task session and resting-state session using correlation map indicating a similar intrinsic network organization. (**A**) Group average of the creative task and resting-state functional connectivity matrices using z-scored Pearson correlation. The brain networks have been represented on the sides. Intra-module functional connections can be seen within the functional systems. The functional connectivity matrix for the overall creative task (on the left), while the resting-state functional connectivity matrix for the overall resting-state session (on the right) represents correlations of intrinsic functional network architecture. (**B**) A standard community detection approach called fast-greedy algorithm (Clauset et al.) was used to partition the overall creative task session and resting-state session into functional brain networks. Each node colour specifies a community. (**C**) Division of the longer creative task session into two halves, i.e. task session 1 and task session 2 and comparison of results with the resting-state session. Network-specific static configuration results for the task session 1, task session 2 and resting-state session represented by functional connectivity matrices and the community detected networks.

All the results of static functional connectivity were reported with a threshold of p < 0.05 false discovery rate (FDR) corrected along with correlation matrices for the overall task and resting-state sessions were obtained as depicted in Fig. 2A. Strong correlations between the brain regions associated with creative cognition during task sessions and resting-state session were compared for static functional interactions during the respective sessions.

For the network specific interactions, an 8 × 8 correlation matrix was obtained for all three sessions. The interaction between various networks during all the three sessions is depicted in Fig. 2C. During all the three sessions, frontoparietal network (FPN) showed stronger connectivity with the salience network (SN), cerebellar network (CN) and the language network (LN) whereas the CN showed stronger connections with the visual network (VN) and visual network showed connectivity with the somatomotor network (SMR) and SN. During task session 1, DMN showed connections with the FPN, LN, SN and CN whereas, during task session 2, the DMN connected with the FPN, LN and CN. During the resting-state session, DMN showed stronger connections with the VN and SMR. The FPN in both task sessions showed connections with the DMN whereas in the resting-state session FPN connected with the VN. The cerebellum in the task session 1 showed connectivity with the DMN, LN and SN whereas in task session 2 FPN connected with the SMR, SN and dorsolateral network (DA). An intrinsic functional network architecture was seen in both the task sessions along with the resting-state session indicating creative cognition during task and at rest.

**Figure 3:**
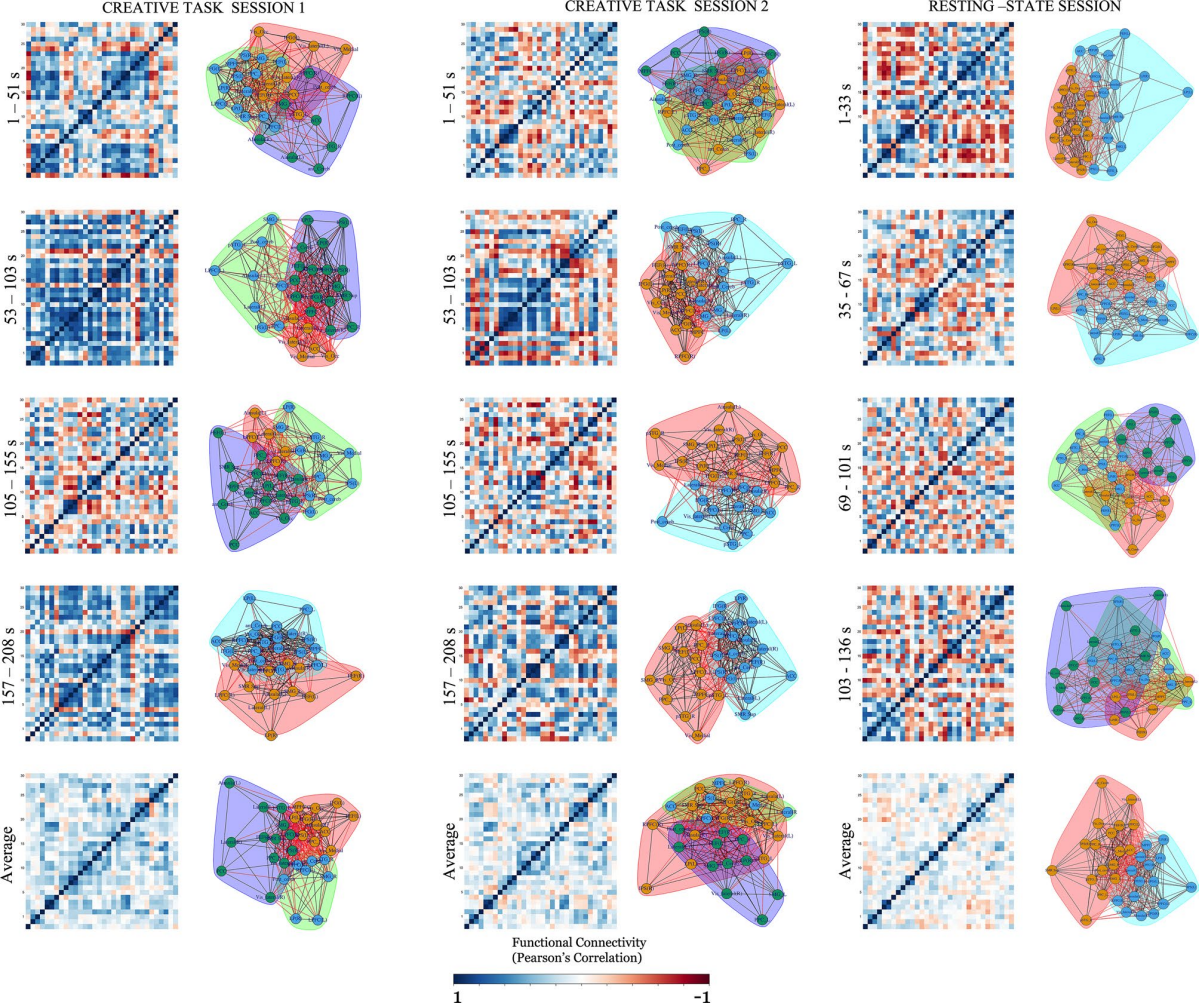
Region-specific changes across time using sliding-window approach (window size = 44 s) for creative task session 1, creative task session 2 and resting-state session. The first column in each session indicates the single windowed correlation map between these times. The bottom row indicates the average of all the first four rows across all the sessions. Second column in every session indicates the graph representation and community detection formed using the fast-greedy algorithm community detection. Each colour indicates community formation for every session, averaged and every windowed correlation map. Node Label List: *mPFC* medial pre-frontal cortex, *LP_L* parietal left (lateral), *LP_R* parietal right (lateral), *PCC* posterior cingulate cortex, *SMR_L* sensorimotor cortex left, *SMR_R* sensorimotor cortex right, *SMR_Superior* sensorimotor cortex (superior), *Visual_Medial* visual cortex (medial), *Visual_Occipital* visual cortex (occipital), *Visual_lateral_L* visual cortex left (lateral), *Visual_lateral_R* visual cortex right (lateral), *ACC* Anterior cingulate cortex, *AInsula_L* anterior Insula left, *AInsula_R* anterior Insula right, *rPFC_L* pre-frontal cortex left (rostral), *rPFC_R* pre-frontal cortex right (rostral), *SMG_L* supramarginal gyrus left, *SMG_R* supramarginal gyrus right, *FEF_L* frontal eye fields left, *FEF_R* frontal eye fields right, *IPS_L* intra-parietal sulcus left, *IPS_R* intra-parietal sulcus right, *LPFC_L* pre-frontal cortex left (lateral), *PPC_L* parietal cortex left (posterior), *LPFC_R* pre-frontal cortex right (lateral), *PPC_R* parietal cortex right(posterior), *IFG_L* inferior frontal gyrus left, *IFG_R* inferior frontal gyrus right, *pSTG_L* superior temporal gyrus left, *pSTG_R* superior temporal gyrus right, *Cereb_Ant* anterior cerebellum, *Cereb_Post* posterior cerebellum.

The region-specific interactions showed slight variations in functional connectivity in the three sessions. The regions of the DMN showed stronger intra-DMN connections along with the bilateral posterior superior temporal gyrus in all the three sessions. During task session 1, the regions of the DMN showed stronger connectivity with the bilateral prefrontal cortex, bilateral posterior parietal cortex, and the right inferior frontal gyrus. The connectivity between the regions of the DMN-bilateral posterior parietal cortex was also seen during task session 2. On the other hand, during the resting-state session observed regions of the DMN having stronger connections with the posterior cerebellum. The FPN showed intra-FPN connectivity across all sessions. During task session 1, the FPN showed stronger connectivity with the regions of the DMN, bilateral prefrontal cortex, right inferior frontal gyrus, left posterior superior temporal gyrus and the posterior cerebellum whereas during task session 2, the FPN showed stronger connections with the left posterior superior temporal gyrus, left inferior frontal gyrus, left lateral parietal cortex and posterior cerebellum. During the resting-state session, the FPN showed connectivity with only the anterior cerebellum. The results of the static configuration were indicative of the role of DMN and FPN and their interaction with other brain networks associated with creative cognition during task session 1 and 2. The resting-state session showed lesser inter-regional connectivity between the DMN, FPN and other networks associated with creative cognition. The cerebellum across all sessions showed intra-cerebellar connectivity and the bilateral prefrontal cortex. During the task session 1, the cerebellum showed stronger connectivity with the visual cortex (occipital and lateral) and bilateral inferior frontal gyrus whereas during the task session 2, the cerebellum showed stronger connectivity with the visual cortex (occipital and lateral) only. During the resting-state session, the cerebellum showed stronger connectivity with the bilateral posterior parietal cortex, visual cortex (lateral, medial and occipital) and left prefrontal cortex. The results of the cerebellar connectivity with the cerebral regions during task sessions 1 and 2 are indicative of the role of cerebro-cerebellar connectivity and the role of the cerebellum in creative cognition. The functional connections are represented by the respective correlation matrices and the networks formed have been depicted in row 5 of Fig. 3 for all the three sessions.

### Task and rest dynamic functional connectivity results

All the results of the dynamic functional connectivity were reported with a threshold of p < 0.05 FDR-corrected. The total number of windows (105 for task and 94 for rest) were divided into four halves and the average of each half was taken and a new window between the time points was constructed as shown in Fig. 3 (row 1, 2 and 3). Similar to the static configuration, the functional connections between brain regions associated with creative cognition during creative task sessions (1 and 2) and resting-state session were compared with the dynamic functional connectivity analysis depicted in row 4 of Fig. 3.

In region-specific interactions, during the first windowed average during task session 1 (i.e. time 1–51 s), the regions of the DMN showed intra-DMN connectivity and with the visual cortex (lateral, medial and occipital), left prefrontal cortex, right posterior parietal cortex, bilateral inferior frontal gyrus and left posterior superior temporal gyrus. The regions of the FPN during this window showed stronger intra-FPN connectivity and with the bilateral parietal cortex, posterior cingulate cortex, lateral regions of the visual cortex, bilateral ipsilateral sulcus, left supramarginal gyrus and superior sensorimotor cortex. The regions of the cerebellum showed stronger connections with the medial and occipital regions of the visual cortex. During the task session 2 (i.e. time 1–51 s), the region of the FPN showed strong connections with the posterior cingulate cortex, lateral parietal cortex, left supramarginal gyrus, right posterior cingulate cortex, right inferior frontal gyrus and bilateral superior temporal gyrus. The cerebellum showed stronger intra-cerebellar connections, left parietal cortex, bilateral posterior parietal cortex and right superior temporal gyrus. For the resting-state session, during the first averaged window (i.e. time 1–33 s), the DMN showed connectivity with the posterior cingulate cortex and occipital and lateral visual cortex. The regions of the FPN showed intra-FPN connections and with superior sensorimotor cortex, right insula, right supramarginal gyrus, bilateral ipsilateral sulcus and anterior cerebellum. The regions of the cerebellum showed strong connectivity with the right prefrontal cortex. The functional connections are represented by the respective correlation matrices and the networks formed have been depicted in row 1 of Fig. 3 for all the three sessions.

For the second windowed average during task session 1 (i.e. time 53–103 s), the DMN showed intra-DMN connections along with superior sensorimotor cortex, lateral visual cortex, right anterior insula, bilateral prefrontal cortex, right supramarginal gyrus, bilateral ipsilateral sulcus, right prefrontal cortex, right posterior parietal cortex, right inferior frontal gyrus, right superior temporal gyrus and anterior cerebellum. The regions of the FPN showed strong connections only with the left parietal cortex. The regions of the cerebellum showed connections with the medial prefrontal cortex and the posterior cingulate cortex. During the task session 2, the regions of the DMN showed strong connections with the right superior temporal gyrus. The regions of the FPN showed stronger intra-FPN connectivity and with the left inferior frontal gyrus, right superior temporal gyrus and posterior cerebellum and the regions of the cerebellum showed intra-cerebellar connections and with the left parietal cortex, left prefrontal cortex and bilateral posterior cingulate cortex. During the resting-state session (i.e. time 35–67 s), the DMN showed connections with the right lateral visual cortex, left parietal cortex and right superior temporal gyrus. The FPN showed connections within the network, medial prefrontal cortex, occipital regions of the visual cortex, right superior temporal gyrus and posterior cerebellum whereas the regions of the cerebellum showed intra-cerebellar connections and with the medial prefrontal cortex, left lateral parietal cortex, superior sensorimotor cortex, occipital regions of the visual cortex, anterior cingulate cortex, right insula, bilateral ipsilateral sulcus, bilateral prefrontal cortex, bilateral posterior cingulate cortex, bilateral inferior frontal gyrus and left superior temporal gyrus. The respective correlation matrices and the networks formed have been depicted in row 2 of Fig. 3 for all the three sessions.

For the third averaged window, during the task session 1 (i.e. time 105–155 s), the regions of the DMN showed connections with the bilateral ipsilateral gyrus. The regions of the FPN were strongly connected with the right supramarginal gyrus, right parietal cortex, right inferior frontal gyrus and right superior temporal gyrus. The regions of the cerebellum showed strong connectivity with the left parietal cortex, superior sensorimotor cortex, left posterior parietal cortex and the left superior temporal gyrus. During the task session 2, the DMN showed connectivity with the left parietal cortex, lateral visual cortex, and right prefrontal cortex. The regions of the FPN showed connections with the occipital visual cortex a left prefrontal cortex whereas the regions of the cerebellum showed intra-cerebellar connectivity and with the left parietal cortex, left lateral and occipital visual cortex, left posterior parietal cortex and left superior temporal gyrus. The DMN regions during the resting-state session (i.e. time 69–101 s) showed interaction with the right insula, right inferior frontal gyrus and anterior cerebellum. The regions of the FPN showed connections with the bilateral prefrontal cortex, posterior cerebellum and right posterior parietal cortex whereas the cerebellum showed connections with the medial prefrontal cortex, right parietal cortex, occipital and lateral visual cortex, bilateral prefrontal cortex and inferior frontal gyrus, anterior cingulate cortex, left superior temporal gyrus and right insula. The respective correlation matrices and the network formed have been depicted in row 3 of Fig. 3 for all the three sessions.

For the final averaged window, during task session 1 (i.e. time 157–208 s), the DMN showed stronger connections with the right supramarginal gyrus, right posterior parietal cortex, right inferior frontal gyrus, bilateral superior temporal gyrus, left parietal cortex and anterior cerebellum. The regions of the cerebellum showed intra-cerebellar connectivity and with the medial prefrontal cortex, left lateral parietal cortex, posterior cingulate cortex, lateral visual cortex, anterior cingulate cortex, bilateral prefrontal cortex, right supramarginal gyrus, bilateral ipsilateral sulcus, right posterior parietal cortex, bilateral inferior frontal gyrus, and bilateral superior temporal gyrus. During the task session 2, the DMN showed connections with the left parietal cortex, right posterior cingulate cortex, bilateral prefrontal cortex, right posterior parietal cortex and right superior temporal gyrus. The regions of the frontoparietal network showed connectivity with the left parietal cortex, posterior cingulate cortex, bilateral prefrontal cortex, bilateral inferior frontal gyrus, right superior temporal gyrus and posterior cerebellum whereas the regions of the cerebellum showed intra-cerebellar connections and with right parietal cortex, posterior cingulate cortex, superior sensorimotor cortex, bilateral visual cortex, right anterior insula, bilateral prefrontal cortex, bilateral ipsilateral sulcus, right posterior parietal cortex and bilateral inferior frontal gyrus. During the resting-state session and final averaged time window (i.e. time 103–136 s), the regions of the DMN showed intra-DMN connectivity and also with the left parietal cortex, lateral, medial and occipital regions of the visual cortex, left ipsilateral sulcus and left posterior parietal cortex whereas the cerebellum showed strong intra-cerebellar connectivity and with occipital visual cortex, anterior cingulate cortex, bilateral prefrontal cortex, right insula, right supramarginal gyrus and left superior temporal gyrus. The respective correlation matrices and the network formed have been depicted in row 4 of Fig. 3 for all the three sessions.

**Figure 4:**
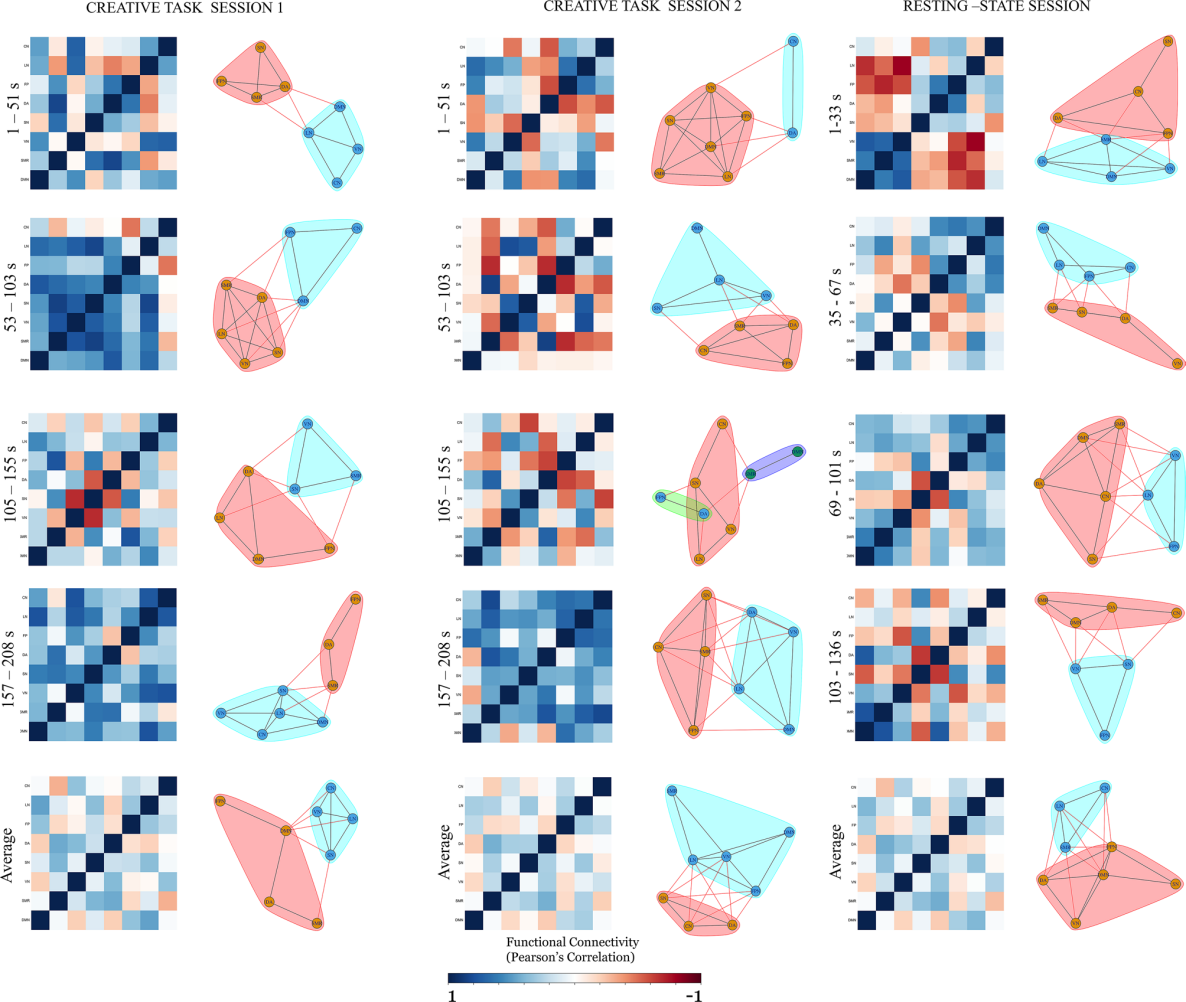
Network-specific dynamic re-configuration of creative brain using sliding window approach (window size = 44 s) for creative task session 1, creative task session 2 and resting-state session. The 32 BOLD time series signals representing 32 ROIs were averaged to form 8 distinct networks. The first column in each session indicates the single windowed correlation map between these times. The bottom row indicates the average of all the first four rows across all the sessions. Second column in every session indicates the graph representation and community detection formed using the fast-greedy algorithm community detection. Each colour indicates community formation for every session, averaged and every windowed correlation map. The edge color here represents the connection within the same community (same edge color of that of the community) or from one community to the other community (edge color of one community indicates the connection from that community to the other). Node Label List: *DMN* default mode network, *FP* frontoparietal network, *SMR* somatomotor network, *DA* dorsal attention network, *SN* salience network, *VN* visual network, *LN* language network, *CN* cerebellar network.

In terms of network specific interactions, in the first averaged window during task sessions 1 and 2 (i.e. time 1–51 s), the DMN showed connectivity with the VN, FPN, CN and LN whereas the cerebellar network showed connections with the DMN, FPN and VN. During the resting-state session (i.e. time 1–33 s) the DMN showed strong connections with the SMR and VN whereas the CN showed only FPN connectivity. In the next averaged window during task sessions 1 and 2 (i.e. time 53–103 s), the DMN showed interaction with the DA, LN and SN whereas the FPN during the task sessions 1 and 2 showed connectivity with the SN and CN. During task session 2, the CN showed connections with the FPN whereas during task session 2, there was no positive connection with the FPN. During the resting-state session (i.e. time 35–67 s), the DMN did not show any interplay with the other networks whereas the FPN had connections with the DA and CN. During the resting-state session showed connectivity of the CN with DA and LN. In the third averaged window, during task sessions 1 and 2 (i.e. time 105–155 s), the DMN showed connections with the DA, LN and SMR whereas the FPN showed connectivity with the SMR and SN during the task session 1, with no strong connections with during the task session 2. The CN showed connections with the LN, SMR during task sessions 1 and 2. During the resting-state session (i.e. time 69–101 s), the DMN showed connections with the SMR, VN, DA, LN and CN whereas FPN showed connectivity with the SN, LN and CN. The CN showed connections with the DMN, SMR, FPN and LN. In the final averaged window, during task sessions 1 and 2 (i.e. time 157–208 s), the DMN showed connections with the LN, FPN, LN and CN whereas the FPN showed connectivity with the VN, LN, SMR, LN and CN. The CN showed connections with the VN, LN, SMR and DA. During the resting-state session (i.e. time 103–136 s), DMN showed interaction with the SMR and DA whereas the FPN showed connectivity with the SN only. The respective network-specific correlation matrices and their connections have been depicted in rows 1–4 of Fig. 4 for all the three sessions. A similar 66 s window used in the dynamic reconfiguration to understand the network-specific interaction has been depicted in the supplementary information (Supplementary Fig. S2).

### Community detection analysis and flexibility results

To examine the evolution of the brain networks associated with creative cognition during the convergent thinking creative task sessions (1 and 2) and resting-state session, a fast-greedy community detection algorithm was applied to the static and dynamic connectivity matrices. All the results of the community detection and network flexibility were reported with a threshold of p < 0.05 (FDR-corrected). The focus of this study was the brain networks associated with creative cognition representing the DMN, FPN and the CN and the regions associated with these networks. The interactions within these regions was also an important focus of this study. Changes in the dynamic functional connectivity of the 8 major functional networks involving various brain regions for various averaged time windows are shown in Fig. 4.

Only two communities were detected in the static configuration associated with creative cognition over the entire scan duration for the whole creative task session as depicted in Fig. 1. After dividing the entire scan duration for task into two sessions i.e. task session 1 and task session 2, we found two communities for task sessions 1 and 2 and for rest as well which is depicted in Fig. 2C. In the static configuration, during task session 1, VN–SN–DA–CN formed communities whereas DMN–SMR–LN–FPN formed communities whereas during the task session 2, DMN–SMR–VN–FPN–LN formed communities and SN–DA–CN formed communities. During the resting-state session, DMN–VN–DA–SN–FPN formed communities whereas SMR–LN–CN formed communities.

For dynamic reconfiguration, in the first averaged window, during task session 1 (i.e. time 1–51 s), the DMN–VN–FPN–CN formed a community whereas SMR–SN–LN–DA formed another community. During task session 2 (i.e. time 1–51 s), CN-DA formed a community whereas other networks formed another community. During the resting-state session (i.e. time 1–33 s), DMN–SMR–VN–LN formed a community whereas SN–DA–FPN–CN formed another community. For the second averaged window, during the task session 1 (i.e. time 53–103 s), the DMN–FPN–CN formed a community and SMR–VN–SN–DA formed another community whereas during task session 2 (i.e. time 53–103 s), DMN–VN–SN–LN formed a community and SMR–DA–FPN–CN formed the other community. During resting-state session (i.e. time 35–67 s), the DMN–FPN–LN–CN formed a community whereas the SMR–VN–SN–DA formed the other community. For the third averaged window, during task session 1 (i.e. time 105–155 s), the DMN–VN–FPN–LN–CN formed a community whereas SMR-SN-DA formed another community. During task session 2 (i.e. time 105–155 s), DMN–SMR formed a community, CN–DA formed other community whereas VN–SN–FPN–LN formed another community. During resting-state session (i.e. time 69–101 s), the DMN–SMR–SN–DA–CN formed a community whereas VN–FPN–LN formed another community. For the final averaged window, during task session 1 (i.e. time 157–208 s), SMR–CN–DA formed a community whereas the other networks formed another community. During task session 2 (i.e. time 157–208 s), DMN–VN–DA–LN formed one community whereas SMR–SN–FPN–CN formed another community. For the resting-state session (i.e. time 103–136 s), DMN–SMR–FPN–VN–CN formed a community whereas other networks formed another community. The community detected network interaction has been depicted in row 1–4 of Fig. 4 for all the three sessions.

The DMN, FPN have been considered as important construct networks for the processes involved during creative cognition. Apart from all these there was variation in the network connections of the salience network which formed communities with the VN and SMR. Further, there was differentiation in the dynamic re-configuration observed with a 66 s window approach and the network interaction have been depicted in the supplementary information (Supplementary Fig. S3).

**Figure 5:**
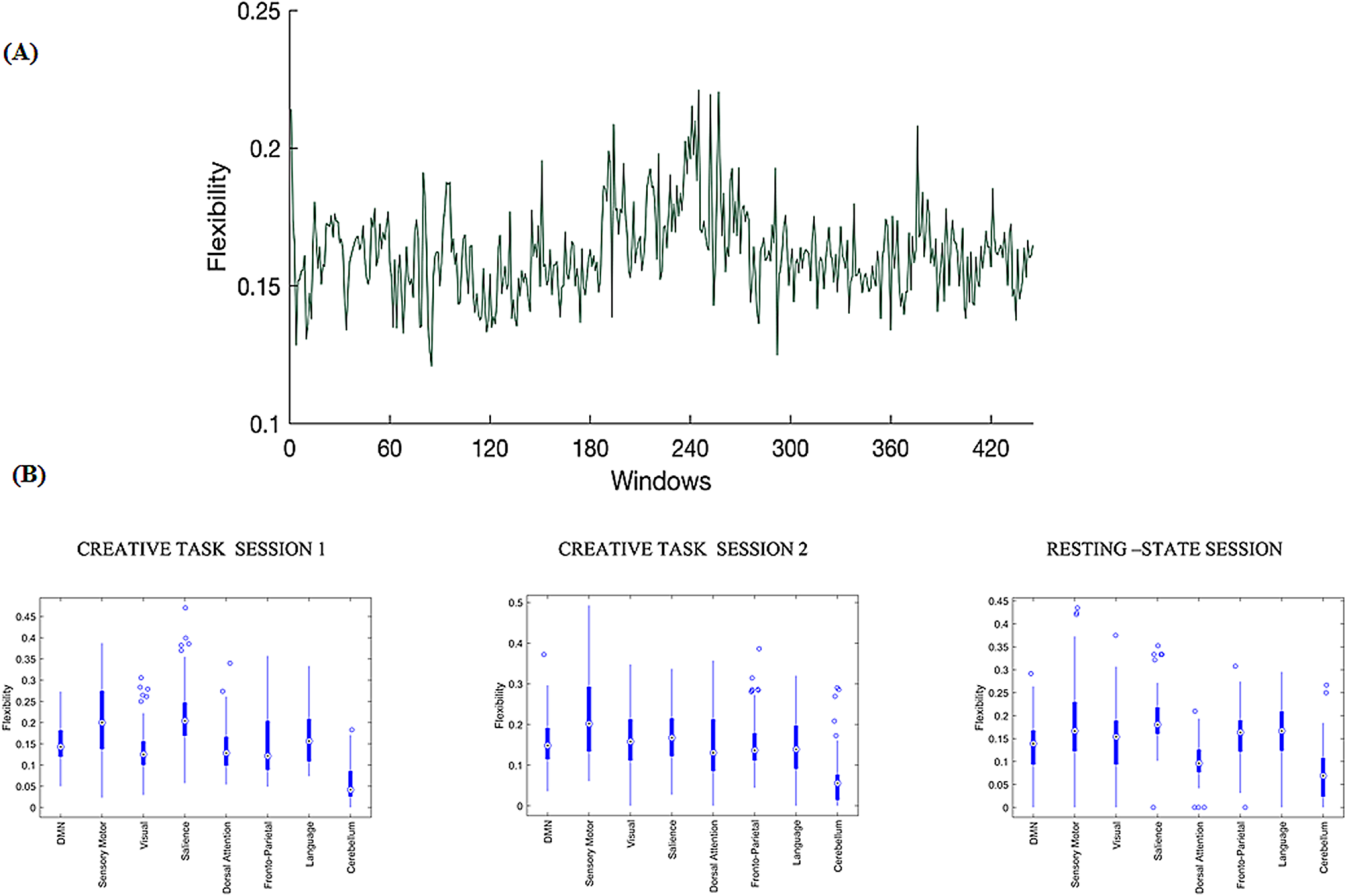
Flexibility of the dynamic brain network associated with creative cognition (**A**) Variation of flexibility across time windows (**B**). Mean flexibility over various networks defined using box plots for creative task session 1, creative task session 2 and resting-state session. Blue circles indicate outliers and blue circles with dots indicate median flexibility value. Uncorrected flexibility values are shown. The networks defined are the default mode network (DMN); sensory/somatomotor network; visual network; salience network; dorsal attention network; fronto-parietal network (FPN); language network; cerebellar network.

Flexibility is an important parameter used to quantify the community evolution of the dynamic brain network in this task-based fMRI study. The variation in flexibility across the dynamic temporal windows is depicted in Fig. 5A. Flexibility in the dynamic network oscillates across the entire time window. Maximum flexibility was observed during the creative thinking task and this increase in flexibility across windows was not modulated by the creative task. We did not find variation in the network flexibility between the CAT and math tasks or across the entire fMRI session. Boxplots indicating variation in the flexibility of the different networks across all sessions, are shown in Fig. 5B. During task session 1, salience network showed highest mean-flexibility [repeated measures ANOVA: F_(8,105)_ = 62.86, p < 0.001] where as somatomotor network showed highest flexibility during task session 2 [repeated measures ANOVA: F_(8,105)_ = 40.39, p < 0.001]. During resting-state session, mean-flexibility was highest in the salience network [repeated measures ANOVA: F_(8,69)_ = 29.301, p < 0.001]. We further compared FPN with other non-frontal regions and during task session 1, FPN showed significant flexibility values with the VN [t_(105)_ = 1.65; p < 0.05] and CN [t_(105)_ = 10.03; p < 0.001] whereas during task session 2, FPN showed significant values only with the CN [t_(105)_ = 10.93; p < 0.001]. During resting-state session, FPN showed significant flexibility with the DMN [t_(69)_ = 2.42; p < 0.01], VN [t_(69)_ = 1.88; p < 0.05], DA [t_(69)_ = 6.66; p < 0.001] and CN [t_(69)_ = 8.47; p < 0.001]. Flexibility of the dynamic brain network associated with both creative task and rest sessions using the 66 s window approach has been depicted in the Supplementary Information (Supplementary Fig. S4).

## Discussion

Network analysis methods in neuroscience help to uncover the coverts processes of cognition. This is achieved by examining the activities of various brain networks involved during various cognitive processes. Creative cognition is a complex cognitive process that involves the engagement of various networks to produce unusual and unique ideas. In this fMRI study, we used a data-driven approach to characterize the neural processes involved during a convergent thinking task that involves the reconfiguration of the large and complex creative networks. Using a network neuroscience approach, we provide evidence of dynamic functional connectivity organized into different communities during creative thinking tasks. The results indicate the activation of the FPN and DMN for creative ideation, as described by previous creativity studies. We also examined the dynamic reconfiguration and interaction and evolution of these brain networks associated with creative cognition. Our results provide support for the evolution and engagement of these brain networks associated with creative cognition which are relevant to distributed brain networks.

Creativity can be categorized into various processes including, but are not limited to, idea generation and retrieval and the combination of remote semantic associations. Creative thinking is a bottom-up process that involves the engagement of the DMN. The combination of semantic associations and their retrieval also follows a bottom-up approach that involves engagement of the DMN and the FPN. Our results extend the prior knowledge of the network neuroscience of creative cognition by revealing the engagement of these two networks, which have been shown by previous studies to be responsible for creative cognition^38,91–93^. Previous creativity studies have identified the medial pre-frontal cortex and posterior cingulate cortex as the core regions of the DMN involved in creative thought generation^35,94,95^. The dynamic association between the DMN and the FPN indicate that the strength of the connections between them is greater during creative cognition. Jung et al.^39^ proposed that interaction between the DMN and the FPN occurs during the production of novel and useful ideas in creative thinking, which is also one of the findings of the present study, in which we tracked the transitions in the network reconfiguration associated with the adaptive nature of the DMN and FPN and examined their role in creative cognition.

Very few neuroimaging studies have examined the role of the subcortical regions such as the cerebellum in cognitive tasks. The cerebellum is considered to play a role in locomotion and co-ordination^96,97^. Some studies have investigated its role in cognitive functions^98–100^. Other studies have suggested that there is connectivity between the cerebellum and the brain regions responsible for creative cognition^101–105^. In this task-based fMRI study, we highlight the role of the cerebellum in creativity using a dynamic connectivity approach based on community detection. Our results reveal interplay between the cerebellum and the DMN during a creative thinking task in what has been referred to as a “hub system” of creative cognition^106^. This interplay between the DMN and the cerebellum explains the role of dynamic brain networks in this task-based fMRI study. Our findings extend the understanding of the role and evolution of the DMN and the cerebellum in convergent thinking tasks.

We also examined network flexibility—a network parameter that has previously been linked with changes in brain networks^17^. Brain network flexibility can be used to track the specific and repetitive changes in networks during a task. The present study also focused on the temporal dynamics of brain networks and tracked the transition of different brain states. Little or no change in flexibility was found across the whole fMRI session during a creative thinking task. In the major networks for creative thinking, the DMN and FPN, greater flexibility was found in the FPN than in DMN during a creative thinking task indicating that the FPN is more likely than the DMN to change its allegiances during combination tasks or a combination of remote semantic association task. The domain-general role of networks, resulting in an increase of flexibility in the FPN, has been reported previously for various tasks^69,107,108^. Our results also suggest the reconfiguration and reorganization of dynamic brain networks. In this specific task-related fMRI study, the communities associated with the frontal network showed strong associations with the posterior regions such as the CN.

To conclude, the present study extends the dynamic role of functional connectivity between various creative regions previously defined by Beaty et al.^35^. Our results corroborated the role of the DMN, which is the hub of creative processes and important for idea generation during a creative thinking task^109^. Further, our findings support the role of the cerebellum during a creative thinking task in a large-scale brain network associated with creative cognition, which was previously found in neuroimaging studies^96,98,99^. We also observed the role of interactions between the DMN and various other networks during the creative thinking task. Using the dynamic network neuroscience approach to reveal the dynamic functional connectivity between various brain networks associated with creative cognition during convergent thinking sheds light on the evolution of brain networks associated with creative cognition.

## Methods

### Participants

Nineteen healthy, young, right-handed adults (8 females; 11 males; mean age = 23 ± 2.28 years; age range 20–29 years) participated in this event-related fMRI study. The participants were asked to provide written consent for their participation. The study was approved by the Institutional Review Board (IRB) of Academia Sinica and National Chiao Tung University, Taiwan. The study was carried out in accordance with the relevant ethical guidelines and regulations. The participants had no history of neurological or psychiatric disorders. They were screened for normal or corrected to normal vision. A written informed consent was given by all participants prior to their participation.

### Psychological measures

Before MRI scanning, all participants underwent a battery of assessments on psychological measures to evaluate their general and specific cognitive functioning. The Chinese version of the Mini-Mental State Examination (MMSE)^110^ was used to assess general cognitive function and the participants showed a mean score of 29.3 ± 0.82. The psychological measures were adapted from the Wechsler Adult Intelligence Scale (WAIS III)^111^, including the subsets of immediate and delayed memory assessment to measure short-term and long-term memory performance, the vocabulary test to assess language knowledge, digit-symbol and letter-number sequencing tests to assess the psychomotor speed, digital span-forward and digital span-backward tests to measure the capacity of working memory, and the arithmetic test to measure quantitative reasoning and ability. The California Verbal Learning Test (CVLT) was used to measure episodic verbal learning and memory performance. The creative quotient was quantified using creativity assessment questionnaire (CAQ)^112^ with the participants showing a mean score of 10.78 ± 8.97. The participants performed digit span-forward and digit span-backward test to assess short term memory^113^ and showed a mean score of 15.42 ± 0.69 and 11.78 ± 2.61 respectively. A letter-number sequencing test was conducted to measure working memory capacity^113^ where the participants showed a mean score of 16.68 ± 3.55.

### Creative fMRI task

The participants performed a modified version of the Chinese version of the remote associates task (CAT), which represents convergent thinking as defined by Huang et al.^74^. In this task, the participants were presented with three Chinese “stimulus words” and were asked to find the “target word,” which was semantically related to the three stimulus words. Before the participants entered the MRI scanner, they took part in practice sessions to understand the kind of test they would be asked to complete. The fMRI experiment consisted of two runs of the CAT, with each run consisting of 14 trials, yielding 28 trials. The experimental paradigm consisted of a 2 s fixation at the start of the experiment followed by a CAT question of 20 s duration. A math problem was provided of 8 s duration after the CAT question, to distract the participants from remembering the previous CAT question. Finally, a jitter of 4 s, 6 s or 8 s (mean = 6 s) was randomly provided for every subject. The duration of each run was 36 s and the experiment consisted of 14 trials of 504 s duration. The participants could press the button only once when they had an answer. An MRI-compatible response box was used to record the button press responses. The responses were verified to meet the CAT demands.

### Imaging data acquisition and pre-processing

Whole-brain imaging data were recorded at the National Yang-Ming University, Taipei, Taiwan using a Siemens 3.0T Magnetron Trio MRI scanner (Siemens, Germany). Using a single-shot T2* weighted echo planar image (EPI) sequence, 252 functional scans were obtained in each run. The image parameters were as follows: TE/TR = 30 ms/2000 ms, flip angle = 90°, Thirty-three contiguous axial slices were acquired with a slice thickness of 4 mm, a 64 × 64 matrix, and a 3.44 mm × 3.44 mm in-plane resolution, FOV = 220 mm. Structural images were acquired with a 3-D ultrafast magnetization prepared rapid acquisition with gradient echo (MPRAGE) imaging sequence with the following parameters: FOV = 256 mm, resolution 256 × 256, slice thickness 1 mm, 192 slices, TR 3500 ms, TE 3.5 ms, TI 1100 ms, and flip angle 7° with no gap between slices. During the fMRI scanning, foam padding and earplugs were used to limit head movement and reduce scanner noise for the subjects.

All pre-processing and denoising of the acquired fMRI data were done using CONN (http://www.nitrc.org/projects/conn) toolbox in MATLAB 2018b (https://matlab.mathworks.com). All the pre-processing steps were performed with the pre-processing pipeline suggested by CONN for volume-based analysis^114^. First, the functional data was realigned and unwrapped for subject motion estimation and correction. Next, the data was slice-time corrected using an ascending interleaved slice order. After realignment, the fMRI data was controlled for all the head motion artifacts. Artifact reduction tools (ART)-based scrubbing was then used to detect and repair the bad volumes using two measures: (1) framewise displacement is greater than 0.9 mm in all directions and (2) global mean intensity threshold is greater than 5 standard deviations from the mean intensity for the entire scan. The artifact reduced functional data was co-registered using an affine transformation to the structural data using the inter-modality co-registration procedure^115,116^ in SPM 12 (Wellcome Department of Imaging Neuroscience, London, U.K.). Then the structural MPRAGE data was normalized into the standardized Montreal Neurological Institute (MNI) space and segmented into white matter (WM), grey matter (GM) and cerebrospinal fluid (CSF) tissue classes using the SPM 12 unified-segmentation and normalization procedure^117^. The indirect normalization in CONN applied this unified-segmentation and normalization procedure to the structural data using the T1 volumes as a reference image and applies similar estimated non-linear transformation to the functional scans. These data are then resampled to the 180 × 216 × 180 mm bounding box with a 2 mm and 1 mm isotropic voxel for functional and structural data respectively using a 4th order spline interpolation. The normalized scans were then smoothened by spatial convolution with an 8-mm full-width half-maximum Gaussian kernel. Apart from the ART-based noise correction, six head motion parameters were obtained from the spatial motion correction and were added for the denoising steps.

To estimate the potential influences of head motion on functional connectivity, the mean head motion for all the participants and the maximum head motion of individuals were estimated using frame wise displacement (FD)^89^ in creative task fMRI and resting-state fMRI data. A series of correlation analysis between the mean FD and the BOLD time series signal across the ROIs were performed.

Moreover, the anatomical component-based noise correction method (CompCor)^118^ was used to perform noise correction in CONN. The anatomical component-based noise correction method (CompCor) includes: (1) Noise correction included components from white matter(WM), cerebrospinal fluid (CSF)^118^—This includes defining potential confounding effects from observed BOLD signal and computed by one-voxel binary erosion to the masks of voxel > 50% in the WM and CSF posterior probability maps. 5 potential noise components are estimated within each area^119^, First component computed as the average BOLD signal and other components computed first four in a PCA of the covariance within the subspace orthogonal to the average BOLD signal and all other potential confounding effects (2) Subject motion parameters^120^—Subject motion parameters consists of 12 potential noise components in order to minimize motion related to BOLD variability which include 3 translation, 3 rotation parameters plus their associated first order derivatives. (3) Identifying outlier scans or Scrubbing^121^—To remove the influence of outlier scans on the BOLD signal a variable number of noise components are used as potential confounding effects. (4) Constant and first-order linear session and constant task effects^114^—Within each session as well as main task, constant linear BOLD signal trends are convolved with a canonical hemodynamic response function (HRF) and are defined as additional noise components in order to reduce the effect of slow trends, initial magnetization transients and constant task-induced responses in the BOLD signal.

### Seed ROI analysis

Thirty-two seed regions of interest (ROIs) were defined in the CONN toolbox, based on those originally defined by ICA analysis based on the Human Connectome Project dataset (http://www.humanconnectomeproject.org) for 497 subjects, like Wolak et al.^122^. The use of publicly available ROI regions in this study would provide a platform of external validation on our findings by other research groups and minimizes bias when the ROIs were selected from our own dataset. The 32 seed ROIs within anatomically and functionally defined cortical and subcortical regions are listed in Table 1 and depicted in the Supplementary Information (Supplementary Fig. S1). For each participant, we estimated individual BOLD time series for the 32 seed ROIs. BOLD signals for each of the individual seed ROIs lasted for 504 s for task run and 180 s for resting-state data.

**Table 1:** Thirty-two seed regions as regions of interest (ROIs) involving 8 major functional brain networks for network configuration.

Hemisphere	ROIs	ROIs representing networks	BA	x	y	z
R	Medial frontal (orbito)	DMN	17	1	55	− 3
L	Middle occipital	DMN	18	− 39	− 77	33
R	Angular	DMN	39	47	− 67	29
R	Precuneus	DMN	7	1	− 61	38
L	Postcentral	SMN	312	− 55	− 12	29
R	Postcentral	SMN	312	56	− 10	29
R	Paracentral	SMN	312	0	− 31	67
R	Calcarine	Visual	17	2	− 79	12
R	Calcarine	Visual	17	0	− 93	− 4
L	Middle occipital	Visual	18	− 37	− 79	10
R	Middle occipital	Visual	18	38	− 72	13
R	Middle cingulate cortex	SN	25	0	22	35
L	Insula	SN	13	− 44	13	1
R	Insula	SN	13	47	14	0
L	Middle frontal	SN	46	− 32	45	27
R	Middle frontal	SN	46	32	46	27
L	Supra-marginal	SN	40	− 60	39	31
R	Supra-marginal	SN	40	62	− 35	32
L	Superior frontal (dorsal)	DA	6	− 27	− 9	64
R	Superior frontal (dorsal)	DA	6	30	− 6	64
L	Inferior parietal	DA	40	− 39	− 43	52
R	Inferior parietal	DA	40	39	− 42	54
L	Middle frontal	FP	46	− 43	33	28
L	Inferior parietal	FP	40	− 46	− 58	49
R	Middle frontal	FP	46	41	38	30
R	Inferior parietal	FP	40	52	− 52	45
L	Inferior frontal (triangular)	LN	45	− 51	26	2
R	Inferior frontal (triangular)	LN	45	54	28	1
L	Superior temporal	LN	22	− 57	− 47	15
R	Superior temporal	LN	22	59	− 42	13
R	Vermis	CN	**–**	0	− 63	− 30
R	Cerebellum	CN	**–**	0	− 79	− 32

*BA* Brodmann area, *CN* cerebellar network, *DA* dorsal attention, *DMN* default mode network, *FP* fronto-parietal, *LN* language network, *SMN* somatomotor network, *SN* salience network, *L* left hemisphere, *R* right hemisphere, *ROIs* regions of interest.

### Task and rest static functional connectivity analysis

Since the creative-task session was longer than the resting-state session, we divided the task session into two equal halves, i.e. creative task session 1 and creative task session 2 (252 s duration each) and compared with the resting-state session (180 s duration), a similar approach used by Denkova et al.^123^ was used in this study.

We examined the static nature of the functional connectivity for the task sessions and the resting-state session. We obtained the BOLD time-series signals for all the participants from 32 ROIs representing 8 known functional networks defined in Table 1. The data for all participants was concatenated and the z-scores were estimated. The Pearson correlation coefficients of the z-scored data was obtained to form 32 × 32 correlation maps for all the three sessions. The correlation maps were further tested for FDR of *p* < 0.05^15,124,125^. Adjacency matrices were formed using graph measures.

### Task and rest dynamic functional connectivity analysis

The time series BOLD signals for all the three sessions were obtained from 32 seed ROIs and were concatenated for the dynamic functional connectivity analysis. To examine dynamic functional connectivity across all the sessions, we used the most used sliding window approach to segment the BOLD signals into time windows. The selection of window length used was 44 s and 66 s with a step of 1TR was based on previous literature which also utilized a window length between 30 and 60 s ^123,126–132^ and also suggesting that, the variability found in this window length (30–60 s), is not found in larger window lengths^126,133^. In the 44 s window length approach, we obtained for 105 windows for the task, session 1 and session 2 each and 69 windows for the resting-state session whereas for the 66 s window length approach, we obtained 94 windows for task sessions each and 58 windows for the resting-state session. We calculated the z-scores followed by the Pearson correlation coefficients for all windows to obtain a 32 × 32 correlation map for every window.

As noted by Achard et al.^124^, not every element in a correlation matrix indicates a functional interaction between different regions. Therefore, it is necessary to compute a statistical false discovery rate (FDR) correction to rate only the significant and true values. We therefore estimated the significant *p*-values for every 32 × 32 window matrix for dynamic reconfiguration indicating the probability of obtaining correlation coefficient values as large as the observed values when there is null true correlation. We obtained the *p-*value matrix by using *t*-tests between the ROIs using the corrcoef function in MATLAB^134^. We further tested the significant *p*-values for FDR of *p* < 0.05^15,124,125^. The final correlation matrix contained only correlations that passed the FDR threshold. Values that did not pass the threshold were set to zero. The FDR based statistical threshold was applied to the 105 windows for task sessions and 69 windows for the resting-state session, irrespective of correlation coefficient being positive or negative. The FDR-corrected correlation matrices for each participant were calculated for the creative task and resting-state sessions. The respective adjacency matrices and community-based networks were obtained for all the windows across all sessions.

To understand the evolution of dynamic functional connectivity across all the 105 windows for both creative task sessions (1 and 2), we obtained a single windowed correlation map between the times 1–51 s, 53–103 s, 105–155 s and 157–208 s. Similarly, to understand the evolution of dynamic functional connectivity across 69 windows for resting-state session, we obtained single windowed correlation maps between the times 1–33 s, 35–67 s, 69–101 s and 103–136 s. Network specific interaction for the dynamic connectivity analysis were also obtained for all windows and sessions. A similar approach was used for the window length 66 s to obtain single windowed correlation maps between the times 1–47 s, 49–95 s, 97–143 s and 145–186 s for task sessions and single windowed correlation maps between the times 1–29 s, 31–59 s, 61–89 s and 91–114 s for resting-state session.

### Network interactions and community detection

Community detection is an approach to decompose a network into sub-networks. In this study, a community detection algorithm was used to understand if communities exist in all the three sessions. The algorithm was applied to the FDR-corrected adjacency matrices in static reconfiguration and dynamic reconfiguration in both creative task session as well as resting-state session. This was done to understand the region-specific interaction during creative task performance as well as during resting-state. We used R software (https://www.r-project.org/) and its integrated development environment R-Studio (https://www.rstudio.com/products/rstudio/) to develop an in-house code for community detection. After the identification of the communities in the dynamic brain networks, the flexibility of the networks was calculated to identify temporal variability of brain networks.

In terms of static interactions, a community detection algorithm was applied to identify communities for the whole brain over the entire scan duration to observe the region-specific changes in the 32 distinct functional brain regions. The community detection algorithm was also applied to understand the role of the 8 distinct functional networks and to observe network-specific changes across all the three sessions.

In terms of dynamic interactions, the FDR-corrected windowed correlation maps between respective times across all the three sessions were then transformed into adjacency matrices to better understand the nature of functional interactions dynamic functional connectivity. For the window length of 44 s, using graph measures the 105 (for each task session) and 69 (for resting-state session), correlation maps were converted to respective adjacency matrices. A community detection algorithm was applied to each of these adjacency matrices across all three sessions to observe the dynamic changes in communities and interaction between 32 ROIs. A similar approach was used to understand the nature of the algorithm on network-specific changes.

The modularity of a network, i.e., the probability of the number of edges that fall in a particular community^85^, was defined using equation I:

1 $$Q = \frac{1}{2m}\sum\nolimits_{ij} {\left[ {A_{ij} - \frac{{k_{i} k_{j} }}{2m}} \right]} \delta \left( {c_{i} ,c_{j} } \right) ,$$

where $A_{ij}$ is edge weight between nodes i and j, $k_{i}$ and $k_{j}$ are sum of the weights of the edges connected to nodes i and j, m is the sum of all of the edge weights in the graph and $c_{i}$ and $c_{j}$ are communities of nodes i and j, $\delta$ is the Kronecker delta function. Higher modularity indicates a higher probability of the number of edges that exist within the community. We calculated the modularity of both static and dynamic reconfiguration of networks. Flexibility is generally defined as the ability to adapt to changes. In a network, flexibility is the number of times that each node changes allegiance, normalized by the total possible number of changes during scan time windows. It is a basic parameter used to understand how network reconfiguration occurs over time and measures the changes in the adherence of one community to another over time^15,17,135^. We sought to understand the flexible nature of the neural processes involved in creative cognition by using a network reconfiguration methodology to determine changes in the communities in brain networks and examine the consistency between static and temporal brain networks. We defined the flexibility across networks as mean-flexibility across all nodes in the network and defined and used in previous literature and adapted from the network community toolbox (http://commdetect.weebly.com/)^15,136,137^:

2 $$F = \frac{1}{N}\mathop \sum \limits_{i = 1}^{N} f_{i}$$

where, $f_{i}$ is the number of times a node changes its community during each session.

## Supplementary Information

Supplementary Information
