# Supplementary material for: Static and dynamic functional connectivity supports the configuration of brain networks associated with creative cognition
Source: Sci Rep. 2021 Jan 8;11:165. doi: 10.1038/s41598-020-80293-2 (PMC7794287; doi:10.1038/s41598-020-80293-2)
Supplement: Supplementary file 1 — Supplementary Information [file 41598_2020_80293_MOESM1_ESM.pdf]

Static and dynamic functional connectivity supports the configuration of brain networks associated with creative cognition

Abhishek Uday Patil[1,2,3], Sejal Ghate[1], Deepa Madathil[1], Ovid J.L. Tzeng[2,3,4,5,6,7], Hsu-Wen Huang[8,9], Chih-Mao Huang[2,3,4]

1. Department of Sensor and Biomedical Technology, School of Electronics Engineering, Vellore Institute of Technology, India
2. Department of Biological Science and Technology, National Chiao Tung University, Taiwan
3. Center for Intelligent Drug Systems and Smart Bio-devices (IDS^{2}B), National Chiao Tung University, Taiwan
4. Cognitive Neuroscience Laboratory, Institute of Linguistics, Academia Sinica, Taiwan
5. College of Humanities and Social Sciences, Taipei Medical University, Taiwan
6. Department of Educational Psychology and Counseling, National Taiwan Normal University, Taiwan
7. Hong Kong institute of Advanced Study, City University of Hong Kong, Hong Kong
8. Research Center for Education and Mind Sciences, National Tsing Hua University, Taiwan
9. Department of Linguistics and Translation, City University of Hong Kong, Hong Kong

Correspondence to:
Chih-Mao Huang, Ph.D.
Address: Department of Biological Science and Technology, National Chiao Tung University, 1001 University Road, Hsinchu, Taiwan
Tel: 886-35712121
E-mail address: cmhuang@nctu.edu.tw

Supplementary Information to “Static and dynamic functional connectivity supports the configuration of brain networks associated with creative cognition”, Chih-Mao Huang.

Methods

Regions of Interests (ROIs)

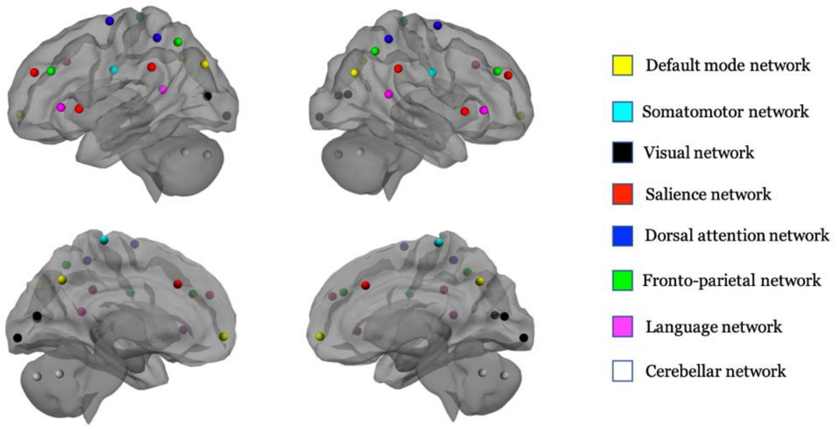

Supplementary Figure 1. Representation of 8 brain network represented by 32 seed ROIs using 3D glass brain. Representation of the 32 ROIs indicating 8 different networks with different colored nodes. The node, their respective networks with the MNI coordinates have been described in the Table 1. Every network has been represented using different colors and has been depicted in the color bar.

Note: The plots defined in the creation of this figure were generated using the MATLAB (R2018b) software (https://matlab.mathworks.com) and Rstudio version 1.2.1335 (https://www. rstudio.com/products/rstudio/).

Results

Supplementary Table 1. Correlations between mean FD, mean power FD and BOLD time series for each ROI for resting-state fMRI session, creative task fMRI 1st session, and creative task fMRI 2nd session

Mean Framewise displacement							Mean power Framewise displacement						
**ROIs**	**resting-state fMRI session**		**creative task fMRI 1st session**		**creative task fMRI 2nd session**		**ROIs**	**resting-state fMRI session**		**creative task fMRI 1st session**		**creative task fMRI 2nd session**	
	**r**	**p**	**r**	**p**	**r**	**p**		**r**	**p**	**r**	**p**	**r**	**p**
**1**	0.16	**0.03***	0.26	**0.00***	-0.10	0.13	**1**	0.19	**0.01***	0.26	**0.00***	-0.10	0.13
**2**	0.16	**0.04***	0.22	**0.00***	-0.09	0.15	**2**	0.18	**0.01***	0.22	**0.00***	-0.09	0.16
**3**	0.15	**0.04***	0.22	**0.00***	-0.09	0.16	**3**	0.18	**0.02***	0.21	**0.00***	-0.09	0.17
**4**	0.15	**0.04***	0.22	**0.00***	-0.08	0.18	**4**	0.17	**0.03***	0.19	**0.00***	-0.08	0.19
**5**	0.14	0.06	0.16	**0.01***	-0.08	0.23	**5**	0.17	**0.03***	-0.16	**0.01***	-0.08	0.20
**6**	0.14	0.06	-0.15	**0.02***	-0.08	0.23	**6**	0.15	0.05	0.15	**0.02***	-0.08	0.24
**7**	0.12	0.10	-0.13	0.04	-0.07	0.26	**7**	0.14	0.06	-0.14	**0.02***	-0.08	0.24
**8**	0.12	0.11	0.12	0.06	-0.07	0.27	**8**	0.14	0.06	0.11	0.08	-0.07	0.28
**9**	0.12	0.11	0.11	0.07	-0.06	0.31	**9**	0.13	0.08	0.10	0.11	-0.07	0.30
**10**	0.12	0.12	-0.11	0.09	-0.06	0.33	**10**	0.13	0.08	0.10	0.11	-0.07	0.30
**11**	0.11	0.13	-0.10	0.11	-0.06	0.33	**11**	0.13	0.08	0.09	0.14	-0.07	0.30
**12**	0.11	0.15	0.10	0.11	-0.06	0.34	**12**	0.13	0.09	-0.09	0.15	-0.06	0.35
**13**	-0.11	0.15	0.09	0.16	-0.05	0.40	**13**	0.12	0.12	0.08	0.21	-0.06	0.39
**14**	0.10	0.20	0.08	0.19	-0.05	0.43	**14**	0.11	0.14	0.07	0.25	-0.05	0.41
**15**	0.08	0.30	0.07	0.24	-0.05	0.44	**15**	0.10	0.18	-0.07	0.27	-0.05	0.42
**16**	0.07	0.35	0.06	0.36	-0.04	0.52	**16**	-0.08	0.27	0.07	0.29	-0.04	0.55
**17**	0.07	0.37	-0.06	0.37	-0.04	0.53	**17**	-0.07	0.34	0.05	0.40	-0.04	0.55
**18**	0.07	0.39	0.05	0.40	-0.04	0.57	**18**	0.07	0.35	0.05	0.41	-0.04	0.56
**19**	-0.06	0.42	0.05	0.41	-0.03	0.61	**19**	0.07	0.38	0.04	0.53	-0.03	0.59
**20**	0.05	0.51	0.05	0.45	-0.03	0.62	**20**	0.06	0.41	0.03	0.59	-0.03	0.60
**21**	0.05	0.54	0.04	0.49	-0.03	0.63	**21**	0.06	0.46	0.03	0.64	0.03	0.60
**22**	-0.04	0.55	0.03	0.63	0.03	0.63	**22**	0.06	0.46	-0.03	0.66	-0.03	0.62
**23**	-0.04	0.60	0.03	0.65	-0.03	0.67	**23**	0.05	0.48	0.02	0.73	-0.03	0.67
**24**	0.03	0.65	0.03	0.66	-0.03	0.68	**24**	0.05	0.49	0.02	0.74	0.03	0.68
**25**	0.03	0.67	-0.03	0.67	0.02	0.71	**25**	0.05	0.54	0.02	0.74	-0.02	0.70
**26**	-0.03	0.67	-0.03	0.68	-0.01	0.83	**26**	-0.04	0.56	-0.02	0.74	-0.01	0.84
**27**	0.03	0.67	0.02	0.70	-0.01	0.87	**27**	0.03	0.65	0.02	0.78	-0.01	0.84
**28**	0.03	0.68	0.02	0.73	-0.01	0.87	**28**	-0.03	0.65	0.01	0.82	-0.01	0.84
**29**	0.01	0.85	0.02	0.80	-0.01	0.91	**29**	-0.02	0.75	0.01	0.89	0.01	0.88
**30**	-0.01	0.88	0.02	0.82	0.01	0.92	**30**	0.02	0.83	0.00	0.95	-0.01	0.90
**31**	0.01	0.92	0.01	0.86	-0.01	0.93	**31**	-0.01	0.85	0.00	0.98	-0.01	0.92
**32**	-0.01	0.93	0.00	0.99	0.00	0.96	**32**	0.00	0.98	0.00	1.00	-0.01	0.94

Note: t-test was used to compare the mean FD and power FD with the BOLD time series for 32 ROIs. Bold *p*-values are significant at $p < .05$

Task and rest dynamic functional connectivity results- Region-specific interaction

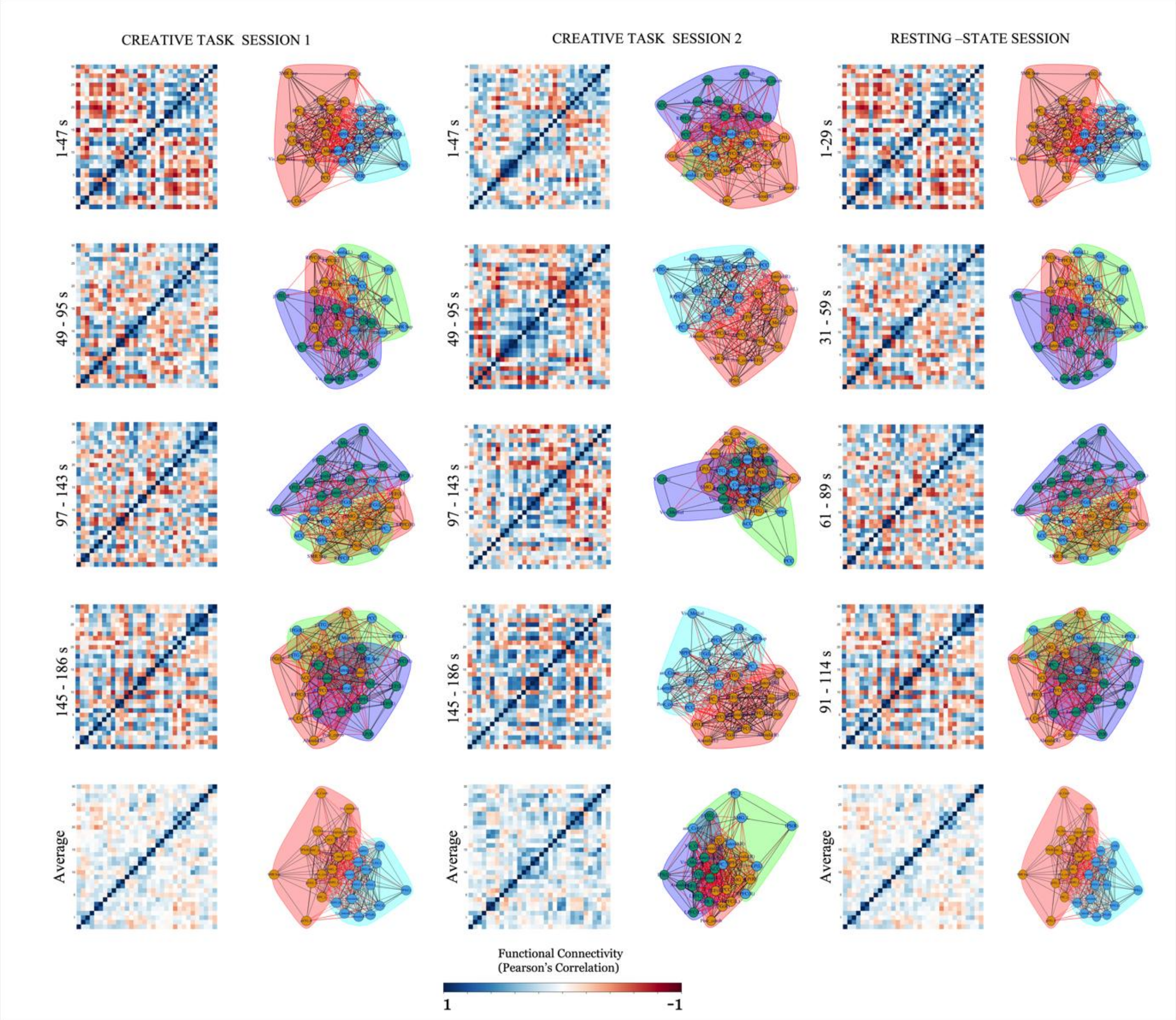

Supplementary Figure 2. Region-specific changes across time using sliding-window approach (window size = 66s) for creative task session 1, creative task session 2 and resting-state session.

The first column in each run indicates the single windowed correlation map between these times. The bottom row indicates the average of all the first four rows for all the runs. Second column in every run indicates the graph representation and community detection formed using the fast-greedy algorithm community detection. Each colour indicates community formation for every run, averaged and every windowed correlation map.

Node Label List: *mPFC*: medial pre-frontal cortex; *LP_L:* Parietal left (lateral); *LP_R:* Parietal right (lateral); *PCC:* Posterior cingulate cortex*; SMR_L:* Sensorimotor cortex left*; SMR_R:* Sensorimotor cortex right*; SMR_Superior:* Sensorimotor cortex (superior)*; Visual_Medial:* Visual cortex (medial)*; Visual_Occipital:* Visual cortex (occipital)*; Visual_lateral_L:* Visual cortex left (lateral)*; Visual_lateral_R:* Visual cortex right (lateral)*; ACC:* Anterior cingulate cortex; *AInsula_L:* Anterior Insula left*; AInsula_R:* Anterior Insula right*; rPFC_L:* Pre-frontal cortex left (rostral)*; rPFC_R:* Pre-frontal cortex right (rostral)*; SMG_L:* Supramarginal gyrus left*; SMG_R:* Supramarginal gyrus right; *FEF_L:* Frontal eye fields left*; FEF_R:* Frontal eye fields right; *IPS_L:* Intra-parietal sulcus left; *IPS_R:* Intra-parietal sulcus right; *LPFC_L:* Pre-frontal cortex left (lateral)*; PPC_L:* Parietal cortex left (posterior)*; LPFC_R:* Pre-frontal cortex right (lateral); *PPC_R:* Parietal cortex right(posterior)*; IFG_L:* Inferior frontal gyrus left*; IFG_R:* Inferior frontal gyrus right*; pSTG_L:* Superior temporal gyrus left*; pSTG_R:* Superior temporal gyrus right*; Cereb_Ant:* Anterior cerebellum*; Cereb_Post:* Posterior cerebellum

Note: The plots defined in the creation of this figure were generated using the MATLAB (R2018b) software (https://matlab.mathworks.com) and Rstudio version 1.2.1335 (https://www. rstudio.com/products/rstudio/).

Task and rest dynamic functional connectivity results- Network-specific interaction

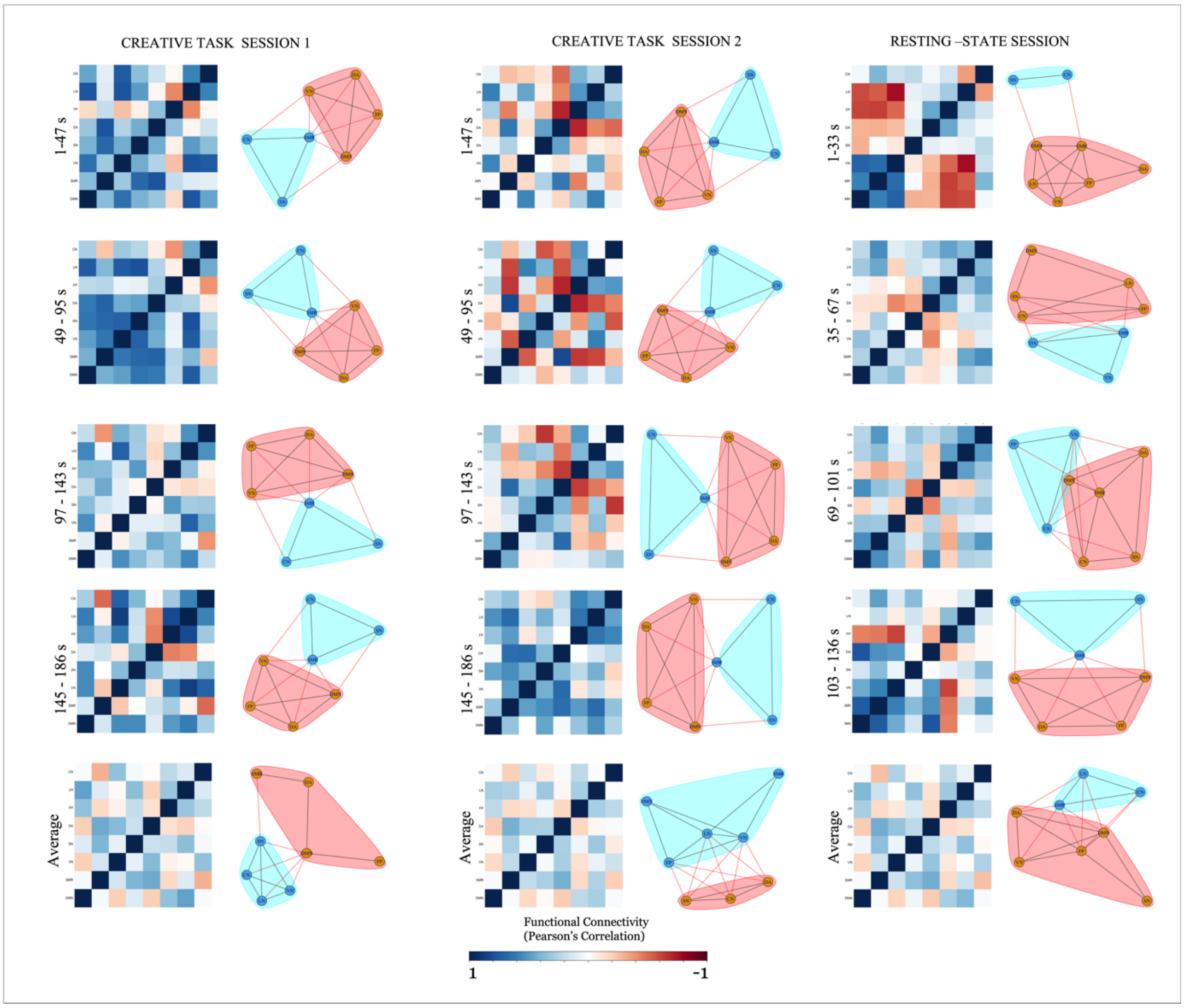

Supplementary Figure 3. Network-specific dynamic re-configuration of creative brain using sliding window approach (window size = 66s) for creative task session 1, creative task session 2 and resting-state session.
The 32 BOLD time series signals representing 32 ROIs were averaged to form 8 distinct networks. The first column in each run indicates the single windowed correlation map between these times. The bottom row indicates the average of all the first four rows for all the runs. Second column in every run indicates the graph representation and community detection formed using the fast-greedy algorithm community detection. Each colour indicates community formation for every run, averaged and every windowed correlation map. The edge color here represents the connection within the same community (same edge color of that of the community) or from one community to the other community (edge color of one community indicates the connection from that community to the other).

Node Label List: *DMN:* Default mode network; FP: Frontoparietal network SMR: Somatomotor Network; DA: Dorsal attention network SN: Salience network; VN: Visual network; LN: Language network; CN: Cerebellar network.

Note: The plots defined in the creation of this figure were generated using the MATLAB (R2018b) software (https://matlab.mathworks.com) and Rstudio version 1.2.1335 (https://www. rstudio.com/products/rstudio/).

Community detection analysis and flexibility results

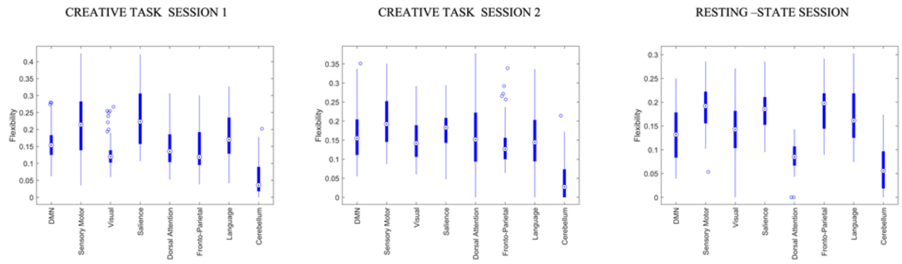

Supplementary Figure 4. Flexibility of the dynamic brain network associated with creative cognition
Mean flexibility over various networks defined using box plots for task run1, task run2 and rest. Blue circles indicate outliers and blue circles with dots indicate median flexibility value.

Note: Uncorrected flexibility values are shown. The networks defined are the default mode network (DMN); sensory/somatomotor network; visual network; salience network; dorsal attention network; fronto-parietal network (FPN); language network; cerebellar network. The plots defined in the creation of this figure were generated using the MATLAB (R2018b) software (https://matlab.mathworks.com).
